# Supplementary material for: A thermodynamic investigation of amyloid precursor protein processing by human γ-secretase
Source: Commun Biol. 2022 Aug 18;5:837. doi: 10.1038/s42003-022-03818-7 (PMC9388646; doi:10.1038/s42003-022-03818-7)
Supplement: Supplementary file 2 — Supplemental Information [file 42003_2022_3818_MOESM2_ESM.pdf]

Supplemental Information

A Thermodynamic Investigation of Amyloid Precursor Protein Processing by Human γ-Secretase

Xiaoli Lu[1,2,3] and Jing Huang[2,3,*]

[1]College of Life Sciences, Zhejiang University, Hangzhou 310058, Zhejiang, China

[2]Key Laboratory of Structural Biology of Zhejiang Province, School of Life Sciences, Westlake University, 18 Shilongshan Road, Hangzhou 310024, Zhejiang, China.

[3]Westlake AI Therapeutics Lab, Westlake Laboratory of Life Sciences and Biomedicine, 18 Shilongshan Road, Hangzhou 310024, Zhejiang, China.

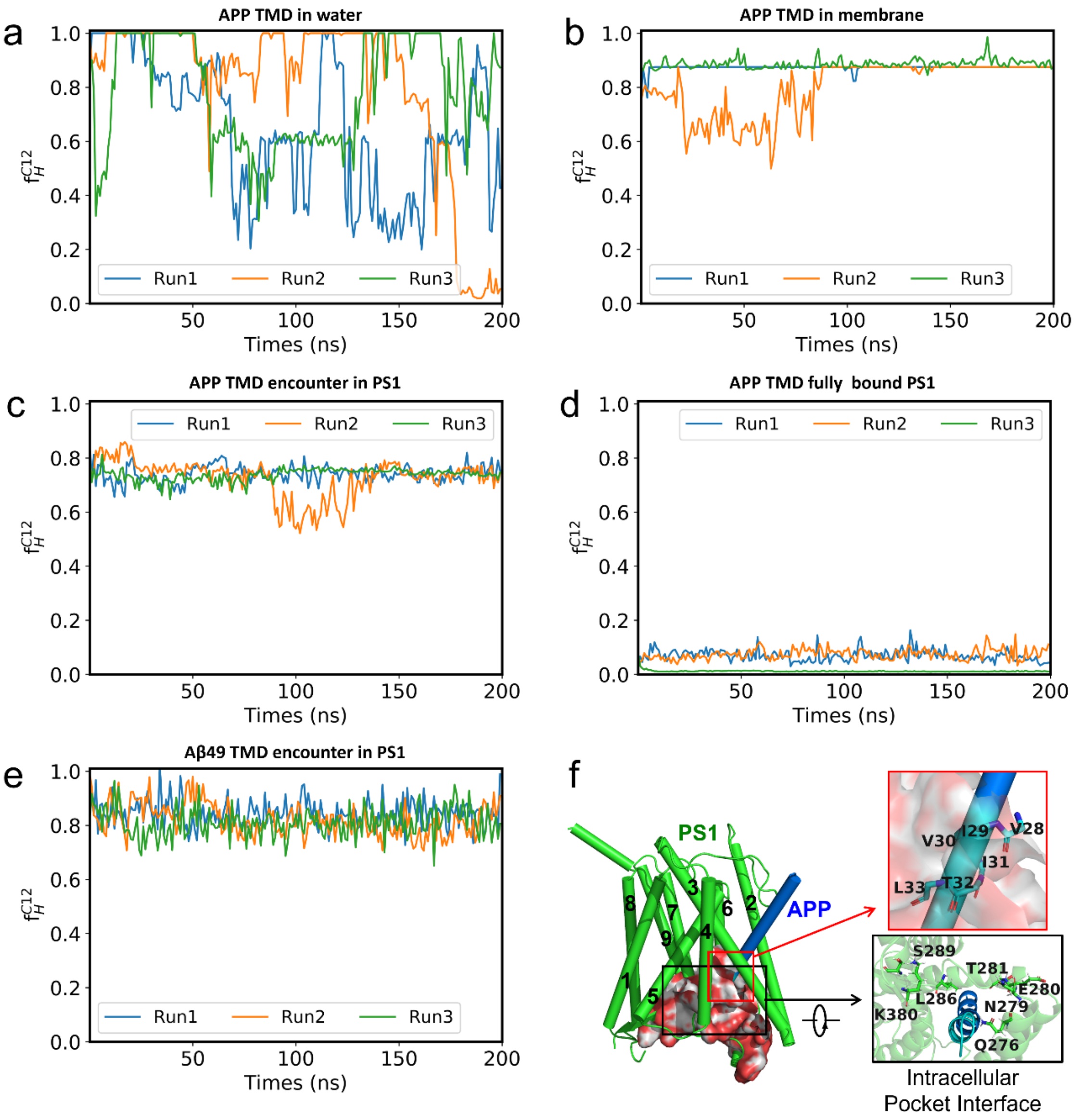

Supplementary Figure 1. Unwinding states of APP TMD in the intracellular pocket of PS1 starting from different initial states. (a-e) Time evolution of fractional residual helicity f_H^{C12} for the C12 region of the APP TMD or Aβ49 TMD during unbiased MD simulations with three replicas under different conditions. (f) The C12 region of the APP TMD is surrounded by water molecules located at the intracellular hydrophilic pocket (electrostatic potential surface) in PS1. The deepest position to which water molecules can penetrate into PS1 is near the V28-I29-V30 region of the APP TMD.

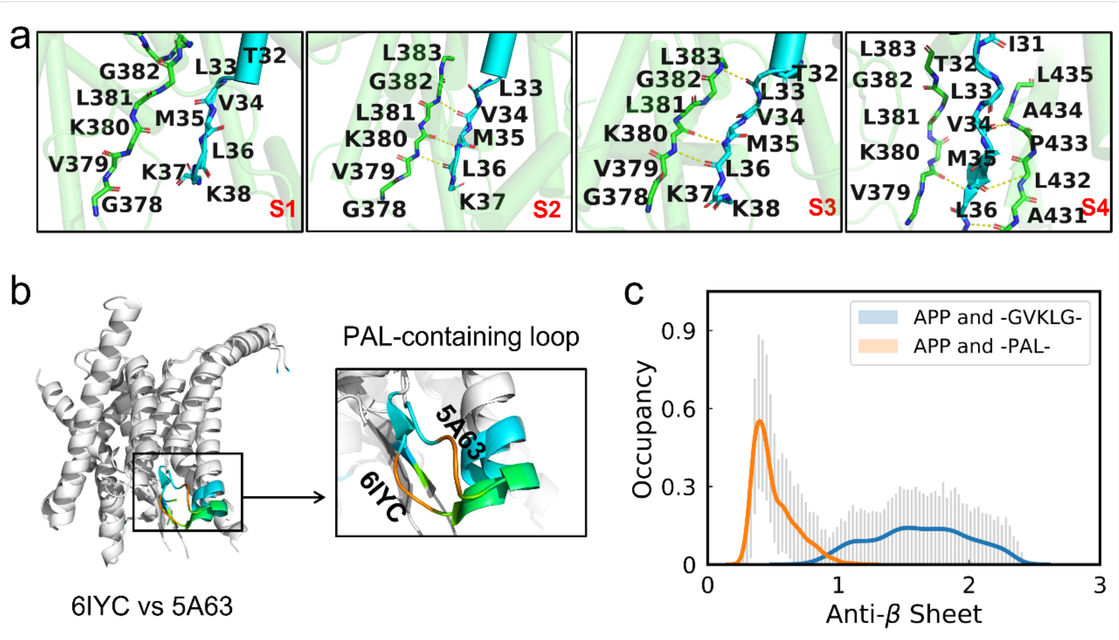

Supplementary Figure 2. The conformations of the GVKLG motif interact with the stepwise unwinding of the TLVMLKKK region of APP TMD. (a) The four sampled intermediate states in the APP TMD (blue) are recognized by PS1 (green) corresponding to S0, S1, S2, S3, and S4. (b) Structural comparison of the PS1 response to the APP-bound state (PDB id: 6IYC) and apo-state (PDB id: 5A63), with the conformational difference of the PAL-containing loop in the two structures highlighted. (c) The occupancy of the anti-parallel β sheet formation between the unwound APP TMD and the GVKLG or PAL motif in 200 ns unbiased MD simulations. Uncertainty is estimated with histogram analysis.

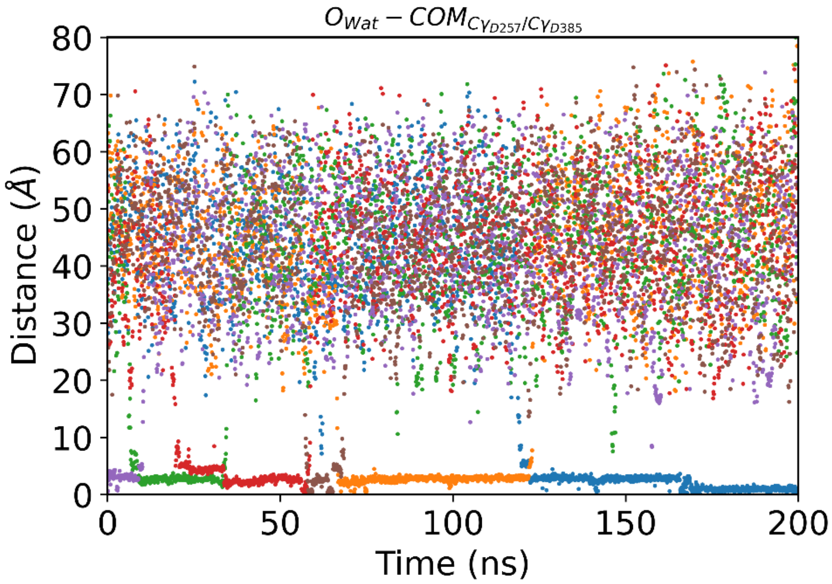

Supplementary Figure 3. Time evolution of the distance between the O atom of water participating in the water bridge (six in total, distinguished by color) and the center of mass (COM) of the Cγ atom of D257/D385.

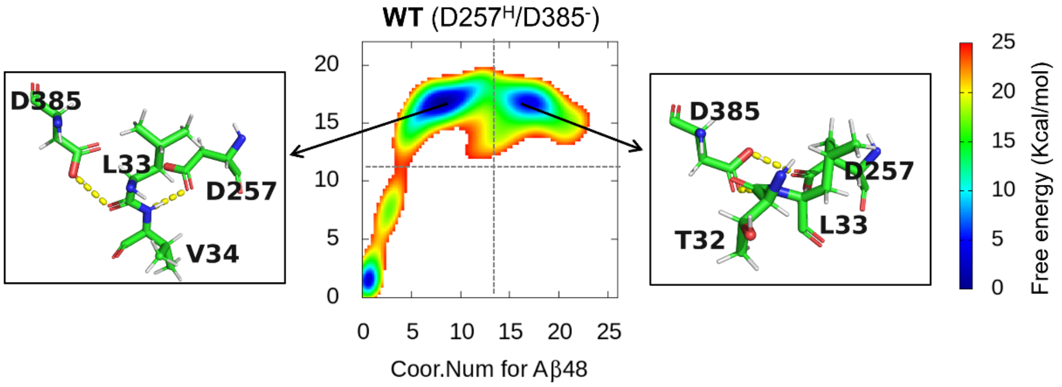

Supplementary Figure 4. 2D free energy profiles of the formation of coordination for Aβ48 and Aβ49 cleavage from the BE-MetaD simulations with $D257^{-}/D385^{H}$.

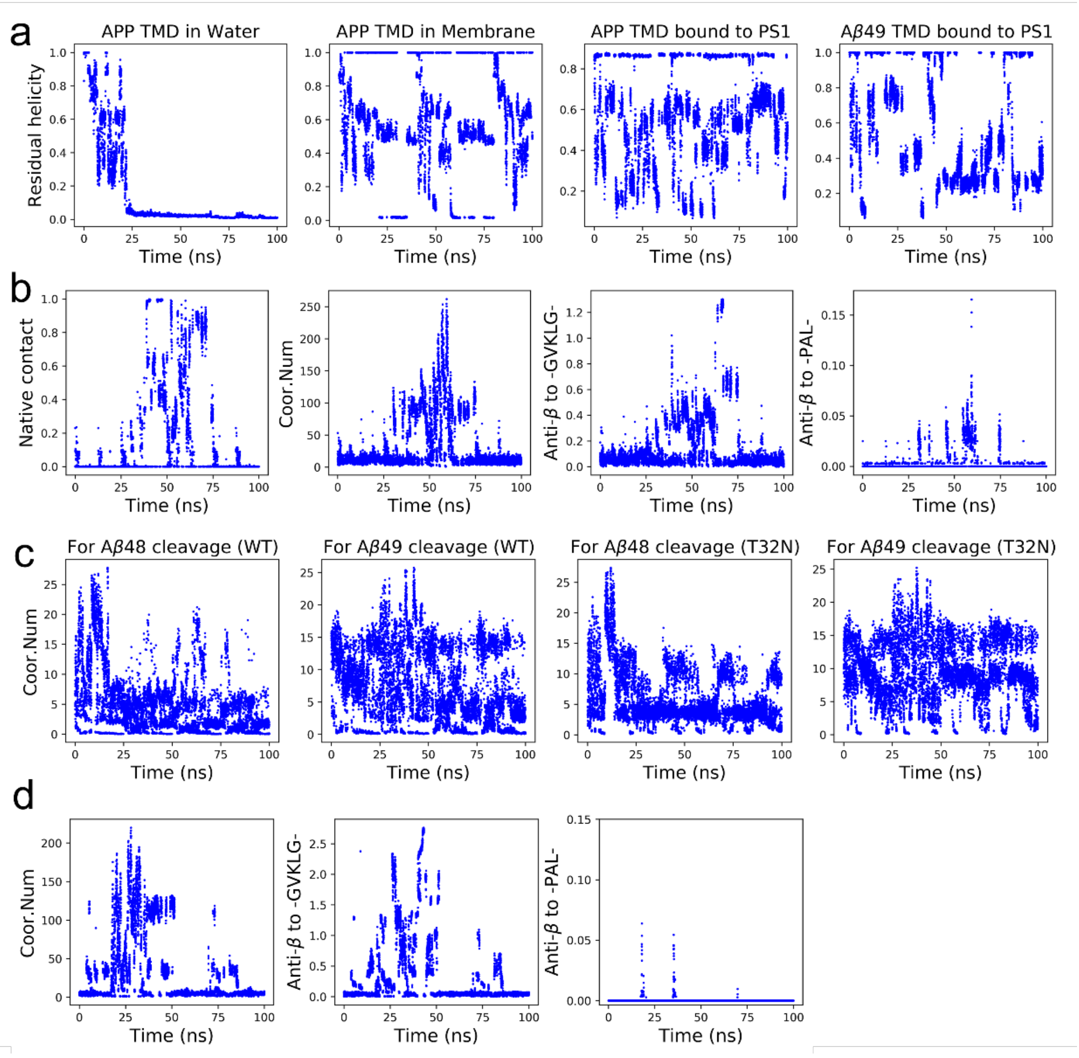

Supplementary Figure 5. Time evolution of the CVs to describe the unwinding state of the substrate TMD and the binding state between the substrate TMD and PS1. (a) Residual helicity of the C12 region of the APP TMD in four simulation systems. (b) Native contact, coordination number, anti-parallel β sheet formation between the unwound APP TMD and the GVKLG and PAL motif in the simulation system of APP TMD bound to PS1. (c) Coordination number between PS1 and scissile bonds in T32-L33 (for Aβ48 cleavage) or L33-V34 (for Aβ49 cleavage) at the catalytic core in the simulation systems of wildtype APP TMD bound to PS1 and T32N mutant APP TMD bound to PS1. (d) Coordination number, anti-parallel β sheet formation between the unwound Aβ49 TMD and the GVKLG and PAL motif in the simulation system of Aβ49 TMD bound to PS1.

Supplementary Table 1. Summary of MD simulations performed on different systems to understand the successive processes of APP TMD recognition by human γ-secretase.

Protein structure	Mimic environment	Dimension (Å)	Simulations (ns)	Targets
α-APP	In water	66*66*66	200 x 3 (unbiased) 200 x 1 (BE-MetaD)	Stability of APP TMD and unwinding process
α-APP	In membrane	60*60*72	200*3 (unbiased) 200*1 (BE-MetaD)	Stability of APP TMD and unwinding process
α-APP-PS1	In membrane	80*80*96	200*3 (unbiased) 200*1 (BE-MetaD)	Stability of APP TMD and unwinding process coupled to PS1 binding
β-APP-PS1	In membrane	80*80*96	200*3 (unbiased) 200*1 (BE-MetaD)	Conformational fluctuations at catalytic core
β-APP(T32N)-PS1	In membrane	80*80*96	200*3 (unbiased) 200*1 (BE-MetaD)	Conformational fluctuations at catalytic core
Aβ49-PS1	In membrane	80*80*96	200*3 (unbiased) 200*1 (BE-MetaD)	Stability of Aβ49 TMD and unwinding process coupled to PS1 binding

Note: α-APP represents APP TMD and is the alpha-helical fold state obtained from PDB 2LLM. β-APP indicates that the APP TMD is in the unwound state extracted from PDB 6IYC. All simulation systems to investigate the conformations of the catalytic core

are established with the unprotonated state and two protonated states ($D257^{H}/D385^{-}$ or $D257^{-}/D385^{H}$).

Supplementary Table 2. Parameters of defined CVs to set up the BE-MetaD simulations of various simulation systems.

CV	Description	Width	Height	Bias-factor	Lower bound	Upper bound
1	Contact	0.1	4	20	0.002	0.998
2	AlphaRMSD	1.0	4	20	0.02	8.0
3	Distance	0.1	2	12	1.6	2.8
4	AntiBetaRMSD	0.2	6	20	0.002	3
5	AntiBetaRMSD	0.1	6	20	0.002	2
6	Coordination number	4	6	40	2	200
7	Coordination number	1	1	20	0.1	25
8	Distance	0.1	0.5	4	0.5	1.1
9	Coordination number	1	1	20	0.1	25
10	Distance	0.1	0.5	4	0.5	1.1
